# When Parents Fail to Mind the Child: Lower Mentalizing in Parents Who Maltreat Their Children

**DOI:** 10.3389/fpsyg.2022.853343

**Published:** 2022-07-12

**Authors:** Anna Maria Rosso

**Affiliations:** Department of Education Sciences, School of Social Sciences, University of Genoa, Genoa, Italy

**Keywords:** parental mentalization, reflective functioning, derogation of attachment needs, dismissing attachment, child maltreatment

## Abstract

Mentalization is considered an essential ability for social cognition as well as a crucial competency in parenting to further the development of internal structures that are decisive for self organization and affect regulation in children. Yet, few empirical studies have investigated whether, and to what extent, parents who maltreat their children poorly mentalize. The aim of this research was to study the mentalization ability in a group of parents who maltreated their children and had been referred by the Courts for Child Custody and Parenting Plan Evaluation (Group 1), and in a comparison, non-clinical group of parents (Group 2). Adult Attachment Interview (AAI), rated in terms of both the Berkeley AAI System and the Reflective Functioning Scale was administered. Group 1 had severely impaired reflective functioning (RF) in 83.3% of cases, whilst impaired RF was found in only 12.5% of Group 2 parents. For the most part, parents in Group 1 showed Negative Reflective Functioning, systematically resisting taking a reflective stance, and the parents who most severely maltreated their children showed distorted and/or self-serving passages associated with a particular type of dismissing pattern of attachment (DS2) based on the derogation of attachment. The frequent occurrence of derogation in these parents likely explains how much the devaluation of relationships and attachment needs, presumably acquired during childhood with defensive purposes and in order to exclude the pain and perception of emotional weakness from awareness, hinders the capacity to care for children in the full respect of their needs.

## Introduction

Individuals who have faced maltreating experiences in their childhood often encounter severe difficulties in parenting their children (for reviews, Morelen et al., 2018; Madigan et al., 2019; Savage et al., 2019), however, considerable variability was observed and research found that not all maltreated individuals become parents who maltreat their children. It was seen that parental mentalization is a protective factor (Berthelot et al., 2015; Ensink et al., 2016; Milan et al., 2021), whilst mentalization deficit was observed in mothers of abused children (Ensink et al., 2017), and in parents who experienced child maltreatment (Berthelot et al., 2015, 2019; Garon-Bissonnette et al., 2021). Adults who underwent child maltreatment are mostly expected to show a distinct disposition to keep away from thinking in mental-state terms. This tendency aims to avoid being in touch with the malevolence of their parents and to prevent facing intense affects and frightening thoughts alone (Fonagy et al., 2002; Allen, 2013). Mentalization deficit, especially concerning the painful attachment experiences faced in childhood, makes the parent more vulnerable to identifying with the aggressor when he/she gets in touch with the troubled child (Fonagy et al., 1993). It was also found that mothers with mentalization deficit exhibit more hostility and intrusiveness in relating to their children (Grienenberger et al., 2005). According to Wang (2021), parents who experienced child maltreatment show a prementalizing mode, which was found to imply the tendency to misread their children’s state of mind and to erroneously attribute malevolent intentions to them (Luyten et al., 2017a,b). Another study (Byrne et al., 2019) also found that parental mentalization deficit can predispose parents to make hostile misattributions about the child’s intentions, which in turn may result in non-accidental injury, physical punishment or emotional abuse. Richey et al. (2016) noted that parents may, for instance, injure the crying or upset child because he/she is persuaded that the child is intentionally and malevolently getting on his/her nerves.

Since mentalization deficit is usually associated with an insecure state of mind regarding attachment, Milan et al. (2021) suggested that mentalizing capacity in individuals who experienced maltreatment may be an index of a more resolved (i.e., secure) attachment state of mind. In line with this hypothesis, Borelli et al. (2015) found that mentalization can be a protective factor against insecure attachment, and that the association between neglect and insecure attachment was more strongly positive among individuals who have lower mentalizing capacity.

The current study aimed to investigate the mentalization ability, the attachment state of mind, and the quality of the childhood experiences in a group of parents who maltreated their children, referred by the Courts for forensic psychological assessment (Child Custody and Parenting Plan Evaluation), and in a comparison non-clinical group of parents. In line with the existing literature, a severe mentalization deficit in parents who maltreated their children associated with an insecure state of mind regarding attachment was hypothesized.

The specific purpose of this study was to explore what kind of childhood experiences regarding attachment, mentalization deficit and insecure attachment pattern are most represented in parents who maltreated their children.

Previous studies found that emotional maltreatment by the parents, rather than physical and sexual maltreatment, had a negative impact both on abused parents and their children (Bottos and Nilsen, 2014). In particular, psychological neglect was found to be associated with later mentalization deficit and maladaptive parenting (Berthelot et al., 2019). Therefore, we expected a replication of these findings.

Concerning mentalizing, the current research is exploratory given that, to our knowledge, no previous study has specifically investigated the type of mentalization deficit in parents who maltreated their children. Regarding attachment, in line with two previous studies (Ammaniti et al., 2004; Hildyard, 2005), we expected a prevalence of insecure attachment with a greater occurrence of the dismissing attachment pattern in these parents.

## Materials and Methods

### Participants

Sixty parents participated in the study: 30 parents who maltreated their children (Group 1) and 30 non-clinical parents (Group 2). Group 1 parents were referred by the Courts for forensic psychological assessment (Child Custody and Parenting Plan Evaluation). They were middle class, aged from 23 to 56 years (*M* = 39.7 years, SD = 8.07), in 66.7% of cases female (*n* = 20), with a mean of 11.83 years of education (SD = 3.19) and in 80% of cases (*n* = 24) were separated or divorced from their spouse after a relationship lasting from 2 to 20 years (*M* = 8.5 years; SD = 4.59). Ten of them (33.3%) (all females) were unemployed. They came from well-off families which allowed them to maintain a good standard of living despite their unemployment status. Regarding this peculiarity of the sample, it is due to the fact that payment for psychological assessment was at their own expense, as per the Court’s policy, therefore it was possible to administer the very time-demanding Adult Attachment Interview (AAI) because they could afford it.

The Court had ascertained severe neglect in 19 cases (63.3%), physical abuse in 8 cases (26.6%) and sexual abuse in 3 cases (10%). The parents were deemed unable to care for their children by the Court and consequently the children were removed from their families and either placed with other family members or, in the majority of cases, placed under the care of Social Services which provided them with a foster family or with residential care.

An independent clinical evaluation was carried out as part of the Expert Witness Report by an expert psychiatrist or clinical psychologist. Only in two cases was a diagnosis on Axis I made, according to DSM-5 criteria. In both cases the mothers suffered from Bipolar II Disorder. In all other cases neither parent had received psychological and/or psychiatric treatment, nor had they been prescribed psychoactive drugs. Seven parents received a diagnosis of Personality Disorder (Borderline Personality Disorders in 2 cases, Histrionic Personality Disorder in 3 cases, Narcissistic Personality Disorder in 1 case, and Paranoid Personality Disorder in 1 case).

The parents in the comparison group were referred to the author by two pediatricians who had been requested to provide referrals of parents they had known for at least 5 years and who presented good parenting skills. Parents of disabled children and parents suffering from psychopathology were excluded. Each pediatrician referred the parents who presented to their outpatient clinics over the following weeks and who fulfilled the established inclusion and exclusion criteria. Thirty-eight of the 52 parents who were asked to participate in the study agreed to be contacted by phone by the researcher. Finally, 30 parents accepted to take part in the study. They were aged between 26 and 45 years (*M* = 39.12; SD = 4.40), females in 56.7% of cases (*n* = 17), in all cases employed and living with their spouse for a mean of 11.67 years (SD = 2.37).

*t*-Test showed no significant differences between the two groups with regard to age (*t* = 0.032, *df* = 58, *p* = 0.975) and education (*t* = 1.588, *df* = 58, *p* = 0.118). They also did not differ significantly with respect to gender (Fisher Exact Test, *p* = 0.256). Regarding occupation, the comparison between the two groups highlighted that distributions were significantly different, with a high percentage of unemployed parents (33.3%) in Group 1 (χ^2^ = 15.41, *df* = 2, *p* = 0.009), whereas in Group 2 all the participants had a stable occupation.

Comparison between the two groups showed significantly longer relationships with the partner parent of the child among the subjects in Group 2 (*t* = −2.162, *df* = 58, *p* = 0.035).

### Measures

Adult Attachment Interview, rated in terms of both the Berkeley AAI System (Main et al., 2003) and the Reflective Functioning Scale (RFS; Fonagy et al., 1998) was administered.

The AAI is a semi-structured hour-long interview designed to classify the state of mind with respect to early attachment experiences. The protocol consists of 18 questions. The interview begins by asking the subject to describe his/her relationship with their own parents in childhood. Then, the subject is requested to provide five adjectives that depict the relationship with each parent and for specific memories that would support the chosen adjectives. The next questions ask the subject to talk about their experiences of emotional distress, physical injury, illness and separation from parents during their childhood. The subject is further asked about possible experiences of rejection, abuse, maltreatment and loss. The interviewee is also asked to talk about his/her opinion regarding the impact of their childhood experiences on their personality and the mental states underlying their parents’ behavior. Finally, the interview shifts to the subject’s current relationship with his/her parents and the present relationship with his/her children, if any. The last question requires the individual to state how experiences of being parented impact on their parenting.

The AAI includes two different sets of subscales: the Scales for Inferred Experiences with Parents and the Scales for Patterned or Organized States of Mind.

For all the Scales for Experiences, the subject’s mother and father are rated separately. Each individual is scored on each of five 9-point scales according to the rater’s best estimate of the parent’s probable behavior during childhood. The five scales take into account maternal and paternal loving, rejecting, involving/role reversing, neglecting and pressuring to achieve behaviors.

The AAI includes nine 9-point scales for assessing relatively patterned or organized states of mind: coherence of transcript, idealization for the parent, insistence upon lack of recall, involved/involving anger, passivity of discourse, fear of loss, dismissing derogation, metacognitive monitoring and overall coherence of mind.

Two additional scales assess unresolved/disorganized states of mind with respect to experiences of loss as well as experiences of abuse (including physical, sexual abuse, and extreme threats) by attachment figures. Disorganization and/or disorientation in thinking or discourse during discussion of a loss or an abuse are indexes of unresolved/disorganized states of mind.

According to the coding system, subjects are classified as “secure/autonomous” if the narrative is sufficiently coherent regardless of the positive or negative quality of the relationships in their childhood. Transcripts are classified as “dismissing” when the subject shows an attempt to minimize the influence of attachment experiences, in particular idealizing or derogating the attachment figures. The category “preoccupied” is assigned to people who appear entangled in their past experiences. They may be confused, passive, vague, fearful, overwhelmed or angry, conflicted and unconvincingly analytical. “Unresolved/Disorganized” is an additional category assigned when the narrative contains markers of lapses in the monitoring of reasoning or discourse during discussion of experiences of loss and/or abuse.

The category “Cannot Classify” is assigned to those transcripts that show a mixture of inconsistent and incompatible states of mind. In the non-clinical populations the latter category is rarely assigned.

Several studies have supported the power of AAI in predicting parenting and subsequent infant-parent attachment (for a review, Bakermans-Kranenburg and van IJzendoorn, 2009).

In the present study, AAI transcripts also allowed to examine the occurrence of the following life-events: childhood experience of abuse, parental loss, institutionalization, illness, severe physical and/or psychiatric illness of a parent, marital separation of the parents and school drop-out.

The RFS was designed to evaluate the capacity of mentalization in the AAI narrative since some questions in the AAI require reflective functioning (RF) (e.g., “Why do you think your parents behaved how they did during your childhood?”), while other questions permit RF (e.g., “Could you describe your first separation from your parents?”).

According to the scoring guidelines, “Awareness of the nature of mental states,” “Explicit effort to tease out mental states underlying behavior,” “Recognizing developmental aspects of mental states,” and “Mental states in relation to the interviewer” are the four markers of RF. After rating each identified passage of the AAI, an overall classification is assigned to the interview ranging from −1 (negative RF) to 9 (exceptional RF).

Validation studies of the RFS (Fonagy et al., 1998) showed discriminant and predictive validity, good inter-rater reliability, low correlations with education level, and no correlation with socioeconomic status and age. In this study no correlation emerged between parents’ RF and their education level (Spearman’s rho = 0.191, *p* = 0.143).

### Procedure

Group 1 parents were met by the author in her private practice. She conducted the psychological assessment on behalf of a clinical psychologist or a psychiatrist Court Expert who carried out the Expert Witness Report. They were administered the measures after a brief “warm-up” interview and after having provided written informed consent to administration of the measures and to using data, after anonymization, for research purposes.

Group 2 parents were interviewed by the author or by undergraduate students in psychology formerly trained in administration by the author through a practicum lasting 3 months which included the administration of at least 10 AAIs. Explanatory letters had been previously given to the parents by their family pediatrician. The author then called the 38 parents who had accepted the pediatrician’s request to be contacted by the researchers. The 30 parents who eventually agreed to participate in the study and gave their written informed consent were scheduled to be interviewed at their home or at the university laboratory.

The approval of the Ethics Committee was not required because when the study was designed the Local Ethics Committee had not yet been established. Even currently in the local institution the request for approval from the Ethics Committee is optional. All the procedures followed in the study were in accordance with Helsinki Declaration of 1975, as revised in 2013, and in conformity with Italian law as established by the National Board of Italian Psychologists’ Code of Ethics.

The AAI protocols, which were initially audio-recorded and later transcribed verbatim, were rated in terms of both the Berkeley AAI System (Main et al., 2003) and the RFS (Fonagy et al., 1998) by the author as well as by an independent rater who was blinded to the group membership. It must be highlighter that both the author and the independent rater had been trained in administration and are certified coding system raters. The inter-rater agreement was assessed with Cohen’s *k* for the AAI overall classification (*k* = 0.86) and using Pearson’s *r* for the Scales of the Experiences and the Scales for State of Mind (*r* ranged from 0.78 for the scale “Idealization of the relationship with the father” to 0.91 for the scale “Coherence of mind”). The inter-rater agreement for the overall classification of RF scale was excellent with *k* = 0.84. All disagreements about overall classifications between the two raters were later discussed and clarified, then the consensus ratings were used.

## Results

### Childhood Experiences

Frequency of childhood experience of abuse, parental loss, institutionalization, illness, severe physical and/or psychiatric illness of a parent, marital separation of the parents, and school drop-out were calculated. Subsequently, the two groups were compared using the χ^2^ non-parametric test for the nominal variables. *t*-Test was used for normally distributed variables and Mann–Whitney test was used for the not normally distributed variables. Data analysis yielded the following results.

No significant differences emerged between the two groups with respect to the following life-events: childhood illnesses, loss of a parent during childhood, severe physical and/or psychiatric illness of a family member during childhood, school drop-out.

Group 1 parents had more experiences of institutionalization during childhood (Fisher Exact Test, *p* = 0.023). Six of them (20%) spent some years in boarding school, while no subject in Group 2 spent long periods far from home during childhood or adolescence.

No significant differences between the two groups emerged regarding any physical abuse suffered during childhood (Fisher Exact Test, *p* = 0.556) or sexual abuse (Fisher Exact Test, *p* = 0.087), nevertheless, four subjects (13.3%) in Group 1 reported sexual abuse, while no abuse occurred to Group 2 parents.

Significant differences emerged between the two groups regarding marital separation of the parents (χ^2^ = 10,472, *df* = 3, *p* = 0.015): 14 (48.6%) parents in Group 1 and 2 (6%) in Group 2 reported marital separation in their family of origin.

### Attachment Patterns and Reflective Functioning

Significant differences (χ^2^ = 42.691, *df* = 3, *p* < 0.001) emerged between the two groups regarding the AAI classification: in Group 1, 93.3% of parents were classified as insecure, whilst in Group 2, 93.3% of parents were classified as secure. In particular, among the parents in Group 1 the Dismissing classification was overrepresented (66.7%) (see Table 1). Twelve out of the 20 dismissing parents were classified DS2 (i.e., using high derogation as a defensive strategy) and 8 were classified DS1 (i.e., using intense idealization as a defensive strategy).

**TABLE 1:** Distribution of AAI classifications.

	Group 1 (*n* = 30)		Group 2 (*n* = 30)	
Attachment pattern	*n*	%	*n*	%
Secure	2	6.7	28	93.3***
Dismissing	20	66.7	2	6.7***
Entangled	3	10	0	0
Unresolved	3	10	0	0
Cannot classify	2	6.7	0	0

***p < 0.0001.

In order to further investigate the differences in the attachment classifications, the differences between the two groups on the scores of the scales regarding experiences and the scales regarding the state of mind were examined.

Concerning the Scales for Experiences, Group 1 had less favorable experiences with regard to maternal and paternal lovingness and more frequently experienced maternal rejection and maternal neglect. On these scales, each transcript is scored according to the rater’s best estimate of the probable behavior of the subject’s parents during childhood. “The judge’s evaluation of the subject’s experiences of parenting will often differ from the subject’s own apparent evaluation” (Main and Goldwyn, 1998, p. 11). Effect sizes were in the large range, with Cohen’s *d* varying from 0.88 for “Neglecting mother” to −1.62 for “Loving mother.” Results are displayed in Table 2.

**TABLE 2:** Descriptive statistics for AAI Scales for Inferred Experiences with Parents.

	Group 1 (*n* = 30)		Group 2 (*n* = 30)					
Experiences	*M*	SD	*M*	SD	*t*	*z*	*p*	*d*
Loving (mother)	2.47	0.77	4.3	1.49	−6.892		<0.0001	−1.62
Loving (father)	2.1	1.09	3.75	1.40	−5.950		<0.0001	−1.33
Rejecting (mother)	3.37	2.19	1.8	1.28	3.811		<0.005	0.90
Rejecting (father)	2.47	1.81	1.83	1.13	2.085		n.s.	
Neglecting (mother)	3.27	2.1	1.75	1.36		3.679	<0.0001	0.88
Neglecting (father)	3.03	2.09	2.42	1.87	1.804		n.s.	
Involving/reversing (mother)	1.8	1.1	1.54	0.93	1.443		n.s.	
Involving/reversing (father)	1.33	0.96	1.2	0.83		0.706	n.s.	
Pressure to achieve (mother)	1.93	1.26	1.75	1.26		1.195	n.s.	
Pressure to achieve (father)	2.07	1.44	2.4	1.86	1.544		n.s.	

*M, mean; SD, standard deviation; p, p-value; d, Cohen’s measure of effect size (|d| < 0.20: negligible; |0.20| < d < |0.50| : small; |0.50| < d < |0.80| : moderate; d > |0.80| : large).*

Regarding the Scales for States of Mind, Group 1 showed higher scores on the scales “Idealization of the relationship with the mother,” “Idealization of the relationship with the father,” “Overall derogation of attachment,” “Insistence on lack of recall,” “Passivity of thought processes,” “Unresolved loss,” “Unresolved trauma,” and “Overall Coherence of the mind.” Effect sizes were in the moderate to large range (Cohen’s *d* ranged from 0.51 for “Passivity of thought processes” to −2.73 for “Overall Coherence of Mind”). A strong and significant difference between the two groups in the predicted direction emerged with respect to RFS (*t* = −8.994, *df* = 52, *p* < 0.0001). Effect size was in the large range (Cohen’s *d* = −2.04). Results are shown in Table 3.

**TABLE 3:** Descriptive statistics for AAI Scales for States of Mind and Reflective Functioning Scale.

	Group 1 (*n* = 30)		Group 2 (*n* = 30)					
State of mind	*M*	SD	*M*	SD	*t*	*z*	*p*	*d*
Idealization (mother)	4.97	2.01	2.63	1.64	5.519		<0.0001	1.28
Idealization (father)	4.47	2.11	2.20	1.53	5.145		<0.0001	1.25
Anger (mother)	2.07	2.29	1.58	1.21		0.794	n.s.	
Anger (father)	1.43	1.13	1.17	0.82		1.036	n.s.	
Derogation (mother)	1.53	1.25	1.21	0.59		0.914	n.s.	
Derogation (father)	1.4	1.22	1	0		1.193	n.s.	
All derogation	1.97	1.52	1.21	0.59		2.351	0.019	0.72
Lack of recall	3.50	1.4	2.33	1.71		4.298	<0.0001	0.75
Passivity	2.73	1.74	2.00	1.10		2.533	0.011	0.51
Unresolved loss	2.40	1.63	1.58	1.01		2.717	0.009	0.62
Unresolved trauma	1.33	0.92	1.00	0		2.052	0.040	0.72
Overall coherence of mind	2.80	1.24	6.25	1.29	−11.144		<0.0001	−2.73

**Reflective Functioning Scale**	1.3	0.92	4.04	1.77	−8.994		<0.0001	−2.04

*M, mean; SD, standard deviation; p, p-value; d, Cohen’s measure of effect size (|d| < 0.20: negligible; |0.20| < d < |0.50| : small; |0.50| < d < |0.80| : moderate; d > |0.80| : large).*

*Reflective Functioning Scale refers to a different measure.*

Then, the two groups were compared with RF scores in the area of “impairment,” defined by Fonagy et al. (1998) as scores below 3. Group 1 showed impaired RF in 83.3% of cases, whilst impaired RF was found in only 12.5% of parents in the comparison group (Fisher’s Exact Test, *p* < 0.0001). Fourteen of the parents who maltreated their children (46.7%) showed an unusual type of deficit of mentalization called “Negative Reflective Functioning,” systematically resisting taking a reflective stance.

## Discussion

The aim of this research was to study the mentalization ability and its association with the state of mind regarding attachment and the childhood attachment experiences in a group of parents who maltreated their children and in a comparison group.

Relative to the family background, we observed that the parents who maltreated their children did not have difficulties deriving from poverty, poor education, lack of a social support network, or physical illnesses, but rather they had more specific difficulties deriving from family backgrounds characterized by high levels of conflict associated with more intense neglecting experiences, especially with their mothers. The analysis of the scales of the AAI concerning experiences indicates significant differences between the two groups regarding experiences of paternal and maternal lovingness, maternal rejection and neglect. These expected results replicated findings from previous studies (Bottos and Nilsen, 2014; Berthelot et al., 2019).

All the parents who maltreated their children but two showed an insecure attachment classification, and in most cases (66.7%) they received a dismissing classification. They had significantly higher scores on the scales of idealization of both parents, global derogation, lack of memories, passivity of thought processes, disorganization relating to experiences of both mourning and trauma, and, finally, significantly lower scores on the dimension of coherence of mind. The marked tendency toward derogation in parents who maltreated their children may specifically explain how much the devaluation of relationships and attachment needs, presumably acquired during childhood with defensive purposes and in order to exclude from awareness the pain and the perception of emotional weakness, hinders the capacity to care for children in the full respect of their needs.

In these parents the dismissing and derogatory state of mind was associated with severe impairment in mentalization. Our results are particularly noteworthy since the average score of the group of these parents is also considerably lower than the scores reported in the literature for clinical samples (e.g., Fonagy et al., 1998; Levinson and Fonagy, 2004).

Reflective capacity was absent in almost all cases, as they mostly provided poor narratives, lacking any reference to mental states. Their answers were extremely evasive, generic, superficial and were characterized by the use of physical or behavioral descriptors and by under-involvement. Furthermore, they often revealed an unusual type of deficit of mentalization, namely Negative Reflective Functioning, systematically resisting taking a reflective stance. It was specifically observed that the parents who most severely maltreated their children showed distorted and/or self-serving passages. They, who themselves often came from a disadvantaged emotional background providing inadequate support, did not manage to retrospectively re-elaborate their own relationship with their parents and retained mental representations shaped by a defensive denial of their own attachment needs and the real shortcomings of their own parental figures. It can be argued that the lack of adequate RF prevented them from attuning themselves with their own children and grasping the extremely negative impact of their neglecting or abusive behavior. The severe impairment of their capacity to conceptualize themselves and their children as being endowed with mental states, beliefs and desires, was likely passed on in an intergenerational fashion, received by their own parents and unmodified by the lack of re-elaboration work.

Findings from the current study offer further support for the need for therapeutic mentalization-based strategies adequately suited to the specific needs of impaired parenting. The prevention policies of welfare services should take into account mentalization deficit, especially if associated with a derogatory state of mind regarding attachment needs, as a major risk factor for failure of parenting. Berthelot et al. (2019) suggested that focusing on mentalization may be crucial for well-timed identification of individuals with a history of child maltreatment who are expecting a child since intervening post-partum with parent-infant dyads may already be a step too late as findings (e.g., Buss et al., 2017) showed that an intergenerational impact of child maltreatment can be observed shortly after birth.

The main limitation of the current study consists in the fact that it does not take into consideration parental RF, as assessed by the Parent Development Interview (Aber et al., 1985), which is aimed at investigating the parent’s mental representation of him/herself as a parent and of the child and their relationship. Future studies, using both AAI and PDI to assess RF would be of interest to investigate the relationship between parental mentalization in different relational contexts (as a child and as a parent) and its influence on parenting, in order to develop more adequate clinical interventions.

Moreover, two further limitations should be pointed out: the two samples were not matched in any way and, in addition, it could be questioned whether the stress involved in the forensic setting may have influenced the willingness of the parents who maltreated their children to reflect on their childhood experiences and whether the insistence on lack of recall may be due to the interview context leading to an overclassification of the dismissing state of the mind. Although the effect of the context cannot be ruled out, the hypothesis that detection of the deficit of the RF is reliable seems more convincing. It must be emphasized that in most cases they showed an absence of RF, and that especially the parents who most severely maltreated their children showed a specific type of deficit through distorted and/or self-serving passages. With regard to the attachment model, the results could also be reliable as the parents who maltreated their children not only showed a tendency to superficiality and idealization that would have led to the DS3 classification, but they also exhibited a noticeable attitude to derogation, and the insistence on lack of recall was especially extreme.

## Data Availability Statement

The raw data supporting the conclusions of this article will be made available by the authors, without undue reservation.

## Ethics Statement

Ethical review and approval was not required for the study on human participants in accordance with the local legislation and institutional requirements. The patients/participants provided their written informed consent to participate in this study.

## Author Contributions

AR designed the study, performed the statistical analyses, and wrote the manuscript. The author confirms being the sole contributor of this work and has approved it for publication.

## Conflict of Interest

The author declares that the research was conducted in the absence of any commercial or financial relationships that could be construed as a potential conflict of interest.

## Publisher’s Note

All claims expressed in this article are solely those of the authors and do not necessarily represent those of their affiliated organizations, or those of the publisher, the editors and the reviewers. Any product that may be evaluated in this article, or claim that may be made by its manufacturer, is not guaranteed or endorsed by the publisher.
